# The association of the ankle-brachial index with incident coronary heart disease: the Atherosclerosis Risk In Communities (ARIC) study, 1987–2001

**DOI:** 10.1186/1471-2261-7-3

**Published:** 2007-01-16

**Authors:** Beth D Weatherley, Jeanenne J Nelson, Gerardo Heiss, Lloyd E Chambless, A Richey Sharrett, F Javier Nieto, Aaron R Folsom, Wayne D Rosamond

**Affiliations:** 1 Duke Clinical Research Institute, Duke University Medical Center, P.O. Box 17969, Durham, NC 27715, USA; 2 Worldwide Epidemiology, MAI-C.2314.2C, GlaxoSmithKline, Five Moore Drive, PO Box 13398, Durham, NC, USA; 3 Department of Epidemiology, School of Public Health, University of North Carolina, Chapel Hill, NC, USA; 4 Department of Biostatistics, School of Public Health, University of North Carolina, Chapel Hill, NC, USA; 5 Department of Epidemiology, Johns Hopkins School of Public Health, Baltimore, MD, USA; 6 Department of Population Health Sciences, University of Wisconsin School of Medicine and Public Health,610 Walnut Street, 707C WARF, Madison, WI 53726, USA; 7 Division of Epidemiology and Community Health, School of Public Health, University of Minnesota, Minneapolis, MN, USA

## Abstract

**Background:**

Peripheral arterial disease (PAD), defined by a low ankle-brachial index (ABI), is associated with an increased risk of cardiovascular events, but the risk of coronary heart disease (CHD) over the range of the ABI is not well characterized, nor described for African Americans.

**Methods:**

The ABI was measured in 12186 white and African American men and women in the Atherosclerosis Risk in Communities Study in 1987–89. Fatal and non-fatal CHD events were ascertained through annual telephone contacts, surveys of hospital discharge lists and death certificate data, and clinical examinations, including electrocardiograms, every 3 years. Participants were followed for a median of 13.1 years. Age- and field-center-adjusted hazard ratios (HRs) were estimated using Cox regression models.

**Results:**

Over a median 13.1 years follow-up, 964 fatal or non-fatal CHD events accrued. In whites, the age- and field-center-adjusted CHD hazard ratio (HR, 95% CI) for PAD (ABI<0.90) was 2.81 (1.77–4.45) for men and 2.05 (1.20–3.53) for women. In African Americans, the HR for men was 4.86 (2.76–8.47) and for women was 2.34 (1.26–4.35). The CHD risk increased exponentially with decreasing ABI as a continuous function, and continued to decline at ABI values > 1.0, in all race-gender subgroups. The association between the ABI and CHD relative risk was similar for men and women in both race groups. A 0.10 lower ABI increased the CHD hazard by 25% (95% CI 17–34%) in white men, by 20% (8–33%) in white women, by 34% (19–50%) in African American men, and by 32% (17–50%) in African American women.

**Conclusion:**

African American members of the ARIC cohort had higher prevalences of PAD and greater risk of CHD associated with ABI-defined PAD than did white participants. Unlike in other cohorts, in ARIC the CHD risk failed to increase at high (>1.3) ABI values. We conclude that at this time high ABI values should not be routinely considered a marker for increased CVD risk in the general population. Further research is needed on the value of the ABI at specific cutpoints for risk stratification in the context of traditional risk factors.

## Background

The ankle-brachial index (ABI) is a simple, non-invasive measure of subclinical atherosclerosis [1]. Prospective studies have found that those with ABI-defined peripheral arterial disease (PAD) are approximately 1.5 to 2 times more likely to have a clinical CVD event than those without PAD [2-4]. The report of the American Heart Association Prevention Conference V concluded that the ABI provides risk information "over and above that provided by traditional risk factors" and suggested that the test "might be a useful addition to the assessment of CHD risk in selected populations...." [1] Recent guidelines for the Detection, Evaluation and Treatment of High Blood Cholesterol in Adults (Adult Treatment Panel III) recommend that an ABI<0.9 be considered a CHD risk equivalent (10-year CHD risk >20%) [5].

One difficulty in implementing guidelines regarding the ABI is the lack of a standardized threshold level. Moreover, in an apparent paradox, several population-based epidemiologic studies, including the Atherosclerosis Risk in Communities (ARIC) study, have found that women have a lower mean ABI which translates to a higher estimated prevalence of ABI-defined PAD than men, despite having lower prevalences of coronary heart disease [6-11]. Women have less intermittent claudication, with a lower prevalence than men demonstrated in five of six population-based epidemiologic studies of the ABI [6-9,12,13], and with a lower incidence in three of four population-based epidemiologic studies [14-17]. Some researchers suggest that gender-specific ABI cutpoints to define PAD might balance the specificity of this diagnosis in women and men [6,18,19].

The risk of CHD as a function of the ABI over its entire range has not been well characterized. The ABI has been found to have a graded, inverse association with CVD morbidity and mortality below ABI values considered "normal"[3,20-22] and to have a U-shaped association with both all-cause and CVD mortality in American Indians [23] and in elderly Americans [24]. A lower incidence of CHD for ABI greater than 1.30 versus ABI 0.90–1.30 was found for African American and white participants of the Atherosclerosis Risk in Communities (ARIC) study [25]. While the association of ABI-defined PAD with CHD risk has been noted for black and white Americans in both the ARIC study [26] and the Cardiovascular Health Study [27], to our knowledge the association of the ABI over its full range with CHD incidence has not been described in these race groups.

We used 13-year follow-up data from the ARIC study to compare the relationship between the ABI and incident CHD between men and women, to estimate the associations between the ABI and incident CHD in African American and white community residents, and to examine the association of the entire range of ABI with incident CHD. Because measurement error may bias these estimates of association, we also explored the effect of ABI measurement error on these estimates.

## Methods

### Study population

The ARIC study cohort consists of 15,792 45–64-year-old members of randomly selected households in four United States communities: Forsyth County, North Carolina; northwest suburbs of Minneapolis, Minnesota; Washington County, Maryland; and black residents of Jackson, Mississippi. Sampling procedures have been detailed elsewhere [28,29]. Each field center's Institutional Review Board approved the study, and each participant gave informed consent at each examination. Participants underwent a baseline clinic examination in 1987–1989, were examined three more times at approximately 3-year intervals, and were interviewed annually by telephone. This report includes follow-up through 2001, with a median of 13.1 years follow-up. Individuals of race/ethnicity other than black or white, and black participants at Minneapolis and Washington County (*n *= 103) were excluded, because numbers are insufficient for analysis. Participants with missing baseline ABI (*n *= 571) or other covariates (*n *= 548) were excluded, as were participants with prevalent CHD (*n *= 687) or unknown baseline CHD status (*n *= 295), leaving for analysis 10028 white and 3650 black participants.

### Ankle-brachial index measurement

During an ultrasound exam, trained and certified sonographers measured ankle and brachial systolic blood pressures with a Dinamap™ 1846 SX automated oscillometric device (Critikon, Inc, Tampa, Florida). They followed a standard protocol, using a contour wrapping technique over the posterior tibial artery at one ankle, selected by the technician to be the right leg if a random number displayed on the computer screen of the work station was even, and the left leg if odd. Two ankle pressure measurements were taken 5 to 8 minutes apart, while the participant was in the prone position before and after undergoing popliteal artery B-mode ultrasound scanning. Measurements of the brachial blood pressures, usually in the right brachial, were subsequently taken approximately every 5 minutes while the participant was in the supine position undergoing ultrasound scanning of the carotid artery. The ABI was then calculated as the average of the two ankle systolic measurements divided by the average of the first two brachial readings. Two participants who had missing ABI because readings that were outside the Dinamap's detection limits (< 30 or > 245 mmHg) were set to missing. To exclude falsely high ABIs due to arterial non-compressibility, 10 values where the ankle pressure was 75 mmHg or more greater than the arm pressure were set to missing; this criterion is reported to have 100% positive predictive value by x-ray for arterial calcification among diabetic patients, although the sensitivity is low [30]. The ABI thus defined ranged from 0.43 to 1.83 in the study population. The reliability coefficient of the ABI based on single ankle and arm oscillometric blood pressure measurements repeated within 1 year apart has been estimated from ARIC data to be 0.61; based on data simulated using ARIC variance estimates, the ratio of the average of two ankle BPs to the average of two arm BPs has a reliability coefficient of approximately 0.70 [31].

### Covariates

Anthropometry, a fasting blood sample, and a medical history were taken at the baseline examination. Lipids were assayed at a central laboratory. Low-density lipoprotein (LDL) cholesterol was calculated using the Friedewald formula [32], and high-density lipoprotein (HDL) cholesterol was measured after dextran-magnesium precipitation of non-HDL lipoproteins. Three seated blood pressure measurements were taken using a random-zero sphygmomanometer and the mean of the last two was used for analysis. Hypertension was defined as sitting systolic pressure ≥ 140 mmHg, diastolic pressure ≥ 90 mmHg, or the self-reported use of antihypertensive medication within two weeks prior to the baseline examination. Diabetes mellitus was defined as a fasting glucose level ≥ 7.8 mmol/L (140 mg/dL), a non-fasting level ≥ 11.1 mmol/L (200 mg/dL), self-reported history of diabetes, or the use of hypoglycemic agents. Intermittent claudication was assessed with the Rose questionnaire [33]. Prevalent CHD was defined as electrocardiographic evidence of a prior myocardial infarction, or a self-reported history of a physician-diagnosed heart attack, coronary bypass surgery, or coronary angioplasty. The mean far-wall carotid intima-medial thickness (IMT) was computed from B-mode ultrasound measures at three 1-cm segments of the extra-cranial carotid artery, bilaterally; maximum likelihood techniques were used to estimate the mean if any of the six measures were missing [34].

### Ascertainment and validation of incident events

Potential incident CHD events were ascertained by annual telephone participant contacts and by surveys of hospital discharge lists and death certificate data for potential cardiovascular events. Trained ARIC personnel abstracted pertinent data from hospital charts, and copied ECGs for central reading. Out-of-hospital deaths were investigated by means of death certificates, interviews with next of kin, and physician questionnaires, with coroner and autopsy reports used when available. All potential clinical CHD events were validated by the ARIC Morbidity and Mortality Classification Committee using published criteria [28]. Twelve-lead ECGs performed every 3 years at ARIC visits through 1998 were reviewed to detect unrecognized myocardial infarction (MI), which was defined by the appearance of a major Q-wave or a minor Q-wave with ischemic ST-T changes, or an MI by computerized NOVACODE criteria [35], confirmed by side-by-side visual comparison of baseline and follow-up ECGs. Hospitalized MI was classified as definite or probable based on chest pain symptoms, cardiac enzyme or marker levels, and ECG changes. Definite fatal CHD was classified based on chest pain symptoms, underlying cause of death, hospital record information, and medical history. A CHD event was defined as a definite CHD death, a validated definite or probable hospitalized MI, or unrecognized MI.

### Statistical analysis

Follow-up time was the time between the first ARIC clinic visit and the first CHD event, or death, last contact date, or December 31, 2001, whichever came first. For an unrecognized MI, the follow-up time was estimated as the midpoint between adjacent visits. Adjusted incidence rates were computed from Poisson regression. Adjusted hazard ratios were computed from Cox regression models. Proportionality of hazards was confirmed by examining Schoenfeld residuals [36]. Continuous covariates were entered as linear terms; nonlinearity was examined using restricted cubic splines [37]. For the ABI, knots for the restricted cubic spline were placed at the 0.05, 0.35, 0.65, and 0.95 ABI quantiles in the overall study population (approximately 0.942, 1.101, 1.193, and 1.346), as recommended [38]. Categorized ABI variables were also examined, including categories commonly used to define peripheral arterial disease (e.g., ABI<0.90). Separate models were constructed for black and white participants, where the covariates' effects were allowed to vary by gender. Analyses were performed using SAS release 8.1 (SAS Institute Inc., Cary, NC). Because regression parameters may be biased when predictors are measured with error [39,40], adjustment for ABI measurement error in Cox regression models was made using a regression calibration technique [41] and assuming an ABI reliability coefficient of 0.70 [31].

## Results

Statistically significant linear associations were found between increasing ABI and gender, race, age, height, smoking status, hypertension, diabetes, HDL- and LDL-cholesterol, and intermittent claudication (Table 1). With increasing ABI, the mean age and LDL-cholesterol, proportions of African Americans and current smokers, and proportions with hypertension, diabetes, and intermittent claudication tended to decrease. HDL-cholesterol and also mean carotid IMT were lower in those with ABI ≤ 0.90. The proportion of men and mean height were higher in those with ABI ≤ 0.80 and also at ABI levels > 1.20.

A total of 686 of the 10028 white participants and 278 of the 3560 African American participants experienced an incident CHD event. In both men and women and in whites and African Americans, CHD incidence was higher for those with ABI-defined PAD than for those without PAD (Table 2), as has been previously reported for ARIC data [26]. White men with PAD, defined as an ABI < 0.90, had an incidence of 21.8 per 1000 person-years, while those without PAD had an incidence of 8.0 per 1000 person-years. Use of multiple ABI categories revealed an increase in CHD incidence with decreasing ABI, from 5.7 per 1000 person-years among white men with an ABI 1.20–1.30 to 24.2 per 1000 person-years for those with an ABI ≤ 0.80. White women with PAD also had a higher CHD incidence (6.9 per 1000 person-years) than those without (3.4 per 1000 person-years). Multiple ABI categories in white women suggested little if any increase in CHD incidence with ABI decreasing from the category 1.20–1.30 (2.3 per 1000 person-years) to 0.80–0.90 (2.9 per1000 person-years), with a notable increase in CHD incidence with an ABI ≤ 0.80 (27.1 1000 person-years). CHD incidence at ABI levels > 1.30 was similar to that estimated for the ABI category 1.20–1.30.

A separate model for African Americans estimated generally higher CHD incidence rates than for whites and similar patterns of increasing CHD risk with decreasing ABI. African American men with PAD, defined as an ABI < 0.90, had a much higher CHD incidence (40.7 per 1000 person-years) than those without PAD (8.9 per 1000 person-years). African American women with PAD had a CHD incidence of 11.4 per1000 person-years, and those without PAD had an incidence of 5.0 per 1000 person-years.

To explore whether gender differences existed in the association of PAD with incident CHD, race-specific hazard ratios (HRs) adjusted for age and field center were estimated from Cox regression models (Table 3). In whites, those with PAD (ABI < 0.90) had twice the hazard of a CHD event than those without, with a HR of 2.81 for men and 2.05 for women; there was no statistically significant effect modification by gender (*P *= 0.39). In African Americans, the HR was higher for men (4.86) than for women (2.34), with marginally statistically significant effect modification by gender (*P *= 0.09). As expected, lowering the ABI cutpoint to define PAD to 0.85 for women increased the estimated HR to 3.31 in white women and 2.76 in black women; the statistical significance of the gender difference in HR decreased in both whites (*P *= 0.69) and blacks (*P *= 0.22).

In both whites and African Americans, modeling the ABI as ordered categories (Table 3) demonstrated a generally increasing CHD hazard with decreasing ABI category. Effect modification of the association of ABI category with incident CHD by gender was statistically significant in whites (*P *= 0.05 whites, *P *= 0.19 blacks), suggesting a gender difference in the shape of the ABI-CHD risk relationship across the spectrum of ABI up to 1.30.

**Figure 1:**
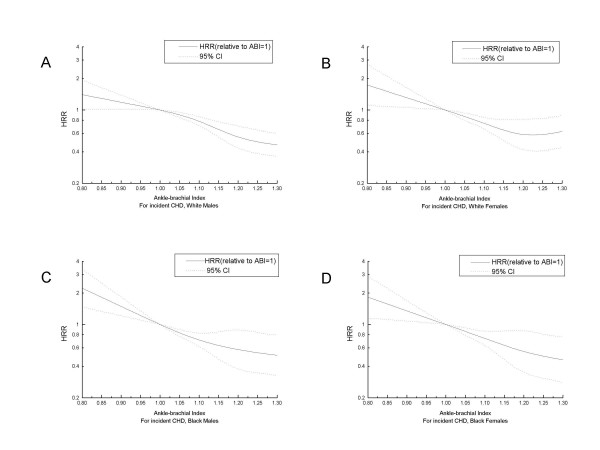
Hazard rate ratio (HRR) relative to an ankle-brachial index of 1.0 for incident coronary heart disease (CHD) by gender for white and African American ARIC participants, 1987–2001. White men (A), white women (B), African American men (C), African American women (D)

Modeling these associations in whites and blacks (Figure 1) as smooth curves using restricted cubic spline functions of the ABI demonstrate generally monotonic increases in CHD risk with decreasing ABI. Wide confidence intervals at ABI values < 0.8 (data not shown) reflect the degree of uncertainty regarding these functions at ABI values of clinical interest. Men and women did not differ significantly regarding the association of ABI, as a continuous spline function, with incident CHD in either whites (*P *= 0.22) or African Americans (*P *= 0.92). Models without the gender-by-ABI interaction showed that the departure from linearity (on the natural log scale) was not statistically significant in either whites (P = 0.12) or blacks (*P *= 0.92). However, in separate models by sex and race, white women did demonstrate a statistically significant non-log-linear relationship (P = 0.04). The figure suggests that the risk of CHD may fail to decline above ABI values of about 1.2 in this population of white women. Modeled as a continuous, linear effect, a 0.10 lower ABI increased the CHD hazard by 25% in white men, by 20% in white women, by 34% in African American men, and by 32% in African American women (Table 4). Adjustment for traditional cardiovascular disease risk factors, excluding hypertension, decreased these hazard ratios by 5 to 9% across race-sex subgroups. The addition of hypertension among the risk factors additionally reduced the estimated HRs by 2% or less. Race-specific estimates of the HRs for a 0.1-unit increase in the ABI, modeled as a linear term, were increased by about 8% in whites and 14% in blacks after adjustment for ABI measurement error in models without covariate adjustment (Table 4). Adjustment for measurement error increased HRs by approximately the same magnitude in models including age and field center as covariates, but had slightly less effect in models additionally including cardiovascular risk factors.

## Discussion

An ABI less than 0.90 is highly sensitive and specific for angiographically-diagnosed PAD and it is now well established that the risk of a clinical cardiovascular disease event is increased in those with ABI-defined PAD. A recent meta-analysis published on behalf of the Ankle Brachial Index Collaboration [42] that included results from six general population studies [4,21,43-46] estimated a relative risk of 1.45 (95% confidence interval 1.08–1.93) for fatal or non-fatal CHD associated with an ABI < 0.9. In the ARIC cohort of 10028 white and 3650 black middle-aged Americans, the estimated age- and field center-adjusted HR associated with an ABI < 0.9 for incident CHD was approximately 2 in all gender-race subgroups except African American men. Results of the ARIC study suggest that the risk of incident CHD may be particularly elevated among black men with an ABI ≤ 0.9 [26], for whom the estimated HR was 4.9 (95% CI 2.8–8.5). These results are of note particularly since African Americans have substantially higher prevalences of PAD and borderline ABI than non-African Americans as reported in previous studies [47-52], a pattern also seen in the ARIC population (e.g., Table 2).

In all gender-race subgroups of the ARIC cohort of middle-aged community residents, the ABI had an approximately log-linear association with CHD risk. This means that not only did the average CHD risk increase exponentially at values < 1.0, but that CHD risk continued to decline at ABI values > 1.0. This is in contrast to the U-shaped association with both all-cause and CVD mortality reported among American Indians in the Strong Heart Study [23] and among elderly Medicare recipients in the Cardiovascular Health Study [24]. Characteristics of ARIC study [25] and Strong Heart Study [23] participants with a high ABI and their CVD risk have been described in detail. Unlike Strong Heart Study participants, ARIC participants with an ABI > 1.4 did not have higher levels of CVD risk factors (e.g., older age, hypertension, triglycerides, LDL-cholesterol) than those with "normal" ABI values; in both studies, the prevalence of diabetes was not higher in those with ABI > 1.4 than in those with "normal" ABI values. While the Strong Heart Study included participants 45–74 years old, and the Cardiovascular Health Study included the elderly (≤ 65 years), ARIC participants were 45–64 years old at baseline. The ARIC cohort represents a comparatively healthy, non-institutionalized, middle-aged population with rates of prevalent PAD, diabetes, and renal disease reflective of those in the general population. Thus, a high ABI in the general population should not be considered as a definite marker for increased CVD risk, as noted previously [25].

The CHD risk for both white and African American women in the ARIC study increased notably at ABI levels < 0.8 and < 0.9 for men, but the small number of events introduces considerable uncertainty regarding the shape of the ABI-CHD association at low ABI levels: very few participants in this middle-aged cohort had an ABI<0.9 (*n *= 381) and, therefore, very few CHD events occurred over 13 years in those with an ABI<0.9 (*n *= 58). There was some evidence that CHD risk failed to decline at higher ABI levels in white women, but this pattern was not evident in the other race-sex subgroups. The lack of a statistically significant gender difference in the association between the ABI and the CHD hazard may have been due, at least in part, to insufficient power. Further analysis of combined studies such as those undertaken by the Ankle Brachial Index Collaboration may allow further exploration of these potential differing associations.

Data from ARIC and other studies suggest that the average risk of future CHD events increases with decreasing ABI as a continuous, but not linear function. Similar results have been reported for exertional leg pain [53], for the subclinical burden of atherosclerosis in the popliteal and carotid arteries [6], and for carotid artery intima-media thickness, and coronary artery calcium [51]. In the Cardiovascular Health Study cohort of older adults, mortality was higher for ABI values above the conventional cutpoint of 0.90, relative to the referent category of 1.11 to 1.20, with variation by age and gender in this association [24]. The choice of a relevant ABI cutpoint at which risk factor modification and therapy should be instituted to reduce future CHD risk is thus unsettled, but should perhaps be based on models of absolute rather than relative risks of future CHD events. PAD is identified as a CHD risk equivalent by the National Cholesterol Education Program (NCEP) Expert Panel [5] and the ABI is a noninvasive screening tool that can be performed readily in an office setting. It is not clear, however, how much information the ABI adds above and beyond that provided by the traditional risk factors, at cutpoint values that can guide clinical practice. Further research should be directed at quantifying the number of persons who would be identified using the ABI who would not have otherwise been identified with traditional risk factors, and the trade-off in the cost of routine screening, with consideration of relevant cutpoint thresholds.

The variability of the ABI measure was previously reported to be approximately 12 percent [54]. Adjustment of ABI as a continuous value for measurement error (i.e., any lack of repeatability of the measurement protocol used in the ARIC study [31]) increased estimated multivariable-adjusted HRs of incident CHD by 8.6% and 5.3% in white men and women, and by 11.0% and 9.2% in African American men and women, respectively. Thus, measurement error appears to attenuate the CHD HR estimates for a 0.1 decrement in the ABI only modestly. However, categorization of a continuous variable measured with non-differential error can result in differential misclassification when the probability of disease is related to the level of the continuous variable [55]. The possible effect of measurement error on the ABI treated as a dichotomous variable, therefore, should be considered when examining ABI-defined PAD, rather than the ABI as a continuous variable, as a predictor.

## Conclusion

African American members of the ARIC cohort had higher prevalences of PAD and greater risk of CHD associated with ABI-defined PAD than did white participants. The risk of CHD increased exponentially with decreasing ABI in African American and white men and women, and the association was similar for men and women in both race groups. Not only did the average CHD risk increase exponentially at values < 1.0 but the risk continued to decline at ABI values > 1.0. Unlike in other cohorts, in ARIC the CHD risk failed to increase at high (>1.3) ABI values. We conclude that at this time high ABI values should not be routinely considered a marker for increased CVD risk in the general population. Further research is needed on the value of the ABI at specific cutpoints for risk stratification in the context of traditional risk factors.

## Competing interests

The author(s) declare that they have no competing interests.

## Authors' contributions

BW, JJN, and GH conceived of and designed the study. BW and LC performed the statistical analyses. BW, JJN, GH, and LC interpreted the results. BW drafted the manuscript. All authors revised the manuscript for intellectual content, and read and approved the final manuscript.

## Pre-publication history

The pre-publication history for this paper can be accessed here:



## Figures and Tables

**Table 1:** Baseline characteristics of participants without prevalent coronary heart disease by ABI category, the ARIC study, 1987–1989*

**Characteristic**	≤ **0.80** **(N = 124)**	**(0.80–0.90]** **(N = 259)**	**(0.90–1.00]** **(N = 1173)**	**(1.00–1.10]** **(N = 3178)**	**(1.10–1.20]** **(N = 4370)**	**(1.20–1.30]** **(N = 3087)**	**>1.30** **(N = 1397)**	**P value**^†^
Male, %	50.0	23.6	22.7	32.8	44.6	54.1	59.1	<0.001
African American, %	44.4	28.6	26.7	27.3	26.8	25.0	22.1	<0.001
Age, y	57.3 (5.2)	54.9 (5.9)	53.6 (5.8)	53.6 (5.7)	53.9 (5.7)	54.2 (5.8)	55.0 (5.6)	<0.001
Height, cm	167.6 (9.0)	164.9 (8.3)	164.4 (8.2)	166.4 (8.7)	168.8 (9.2)	170.3 (9.4)	171.1 (9.5)	<0.001
Smoking status, %								
Current smoker	62.9	43.6	32.3	27.1	24.8	22.7	20.8	<0.001
Former smoker	24.2	22.4	27.1	29.3	32.3	35.1	32.5	
Never smoker	12.9	34.0	40.6	43.6	42.9	42.2	46.7	
Hypertension, %	64.5	41.3	37.2	35.6	32.9	29.2	29.7	<0.001
Diabetes, %	24.2	13.5	10.2	8.6	8.4	7.5	8.6	<0.001
HDL-C, mmol/L	1.22 (0.39)	1.35 (0.45)	1.38 (0.44)	1.40 (0.45)	1.36 (0.45)	1.32 (0.43)	1.30 (0.42)	<0.001
LDL-C, mmol/L	3.99 (1.06)	3.67 (1.17)	3.58 (1.03)	3.56 (1.04)	3.52 (1.01)	3.51 (0.98)	3.53 (0.98)	0.017
Carotid IMT, mm^§^	0.94 (0.32)	0.79 (0.23)	0.73 (0.20)	0.72 (0.19)	0.71 (0.17)	0.71 (0.16)	0.72 (0.17)	0.47
Rose intermittent claudication, %	11.4	3.1	1.2	0.6	0.5	0.1	0.4	<0.001

*Values are mean (SD) unless otherwise noted. ARIC indicates Atherosclerosis Risk in Communities; HDL-C, HDL-cholesterol; LDL-C, LDL-cholesterol; ABI, ankle-brachial index; IMT, intima-medial thickness.

^†^For the test of no rank correlation.

**Table 2:** Sample size, number of incident CHD events, and age- and field-center adjusted incidence rates (per 1,000 person-years) by gender, race, and ABI level: the ARIC study, 1987–2001*

	**Men**			**Women**		
	
**Covariate**	**Sample size**	**CHD events**	**Rate (95% CI)**	**Sample size**	**CHD events**	**Rate (95% CI)**
			**Whites**			
PAD						
ABI < 0.90	80	19	21.8 (13.9 – 34.3)	172	14	6.9 (4.1–11.6)
ABI ≥ 0.90	4446	431	8.0 (7.3–8.9)	5330	222	3.4 (3.0–3.9)
ABI < 0.90 men, < 0.85 women	80	19	21.9 (13.9–34.4)	69	9	11.1 (5.8–21.3)
ABI ≥ 0.90 men, ≥ 0.85 women	4446	431	8.0 (7.3–8.9)	5433	227	3.4 (3.0–3.9)
ABI						
≤ 0.80	38	10	24.2 (13.0–45.0)	31	9	27.1 (14.0–52.2)
(0.80–0.90]	43	9	19.5 (10.1–37.6)	142	5	2.9 (1.2–7.0)
(0.90–1.00]	195	27	11.9 (8.2–17.4)	665	35	4.6 (3.3–6.3)
(1.00–1.10]	764	91	10.4 (8.4–12.8)	1546	71	3.9 (3.1–4.9)
(1.10–1.20]	1489	169	9.7 (8.3–11.2)	1712	66	3.1 (2.5–4.0)
(1.20–1.30]	1321	92	5.7 (4.6–7.0)	994	30	2.3 (1.6–3.3)
>1.30	676	52	5.9 (4.5–7.8)	412	20	3.5 (2.3–5.5)
			**African Americans**			
PAD						
ABI < 0.90	42	14	40.7 (24.0–69.0)	87	11	11.4 (6.3–20.7)
ABI ≥ 0.90	1308	131	8.9 (7.5–10.6)	2123	122	5.0 (4.2–5.9)
ABI < 0.90 men, < 0.85 women	42	14	40.7 (24.0–69.0)	52	8	13.6 (6.8–27.2)
ABI ≥ 0.90 men, ≥ 0.85 women	1308	131	8.9 (7.5–10.6)	2158	125	5.0 (4.2–6.0)
ABI						
≤ 0.80	24	6	30.9 (13.8–69.3)	31	7	23.7 (11.3–49.9)
(0.80–0.90]	18	8	52.5 (26.2–105.2)	56	4	6.0 (2.2–16.0)
(0.90–1.00]	71	8	10.5 (5.2–21.0)	242	20	7.6 (4.9–11.8)
(1.00–1.10]	278	34	11.6 (8.3–16.2)	590	46	6.9 (5.2–9.3)
(1.10–1.20]	461	42	8.3 (6.2–11.3)	708	31	3.8 (2.6–5.3)
(1.20–1.30]	349	36	8.7 (6.2–12.0)	423	19	3.9 (2.5–6.1)
>1.30	149	11	6.2 (3.4–11.2)	160	6	2.9 (1.3–6.5)

*Rates are presented at age 55. CHD indicates coronary heart disease; ABI, ankle-brachial index; ARIC, Atherosclerosis Risk in Communities; CI, confidence interval; PAD, peripheral arterial disease.

**Table 3:** Age- and field-center-adjusted CHD incidence hazard ratios for various ABI categories from Cox models for white and African American participants: the ARIC study, 1987–2001*

	**Whites**		**African Americans**	
	
**Covariate(s)**	HR*Men (95% CI*)	**HR Women (95% CI)**	**HR Men (95% CI)**	**HR Women (95% CI)**
PAD				
ABI < 0.90	2.81 (1.77–4.45)	2.05 (1.20–3.53)	4.86 (2.78–8.47)	2.34 (1.26–4.35)
ABI ≥ 0.90	1.00 (reference)	1.00 (reference)	1.00 (reference)	1.00 (reference)
ABI < 0.90 men, < 0.85 women	2.81 (1.77–4.45)	3.31 (1.70–6.45)	4.86 (2.79–8.47)	2.76 (1.35–5.66)
ABI ≥ 0.90 men, ≥ 0.85 women	1.00 (reference)	1.00 (reference)	1.00 (reference)	1.00 (reference)
ABI				
≤ 0.80	2.39 (1.24–4.60)	7.19 (3.59–14.41)	2.80 (1.16–6.73)	3.60 (1.62–8.00)
(0.80–0.90]	1.94 (0.97–3.84)	0.76 (0.31–1.89)	4.87 (2.25–10.56)	0.87 (0.31–2.41)
(0.90–1.00]	1.15 (0.75–1.77)	1.18 (0.79–1.77)	0.91 (0.42–1.96)	1.10 (0.65–1.86)
(1.00–1.10]	1.00 (reference)	1.00 (reference)	1.00 (reference)	1.00 (reference)
(1.10–1.20]	0.93 (0.72–1.20)	0.81 (0.58–1.13)	0.71 (0.45–1.12)	0.54 (0.34–0.85)
(1.20–1.30]	0.54 (0.41–0.73)	0.60 (0.39–0.92)	0.74 (0.46–1.18)	0.56 (0.33–0.95)
> 1.30	0.57 (0.40–0.80)	0.90 (0.55–1.49)	0.53 (0.27–1.04)	0.42 (0.18–0.98)

*CHD, coronary heart disease; ABI, ankle-brachial index; ARIC, Atherosclerosis Risk in Communities; HR, hazard ratio; CI, confidence interval; PAD, peripheral arterial disease.

**Table 4:** CHD hazard ratios estimated from Cox regression for a decrement of 0.10 in ABI modelled as a linear, continuous term: uncorrected and corrected for ABI reliability

	**Whites**				**African Americans**			
	**Men**		**Women**		**Men**		**Women**	
	
**Covariates**	**Uncorrected HR** **(95% CI)**	**Corrected HR** **(95% CI)**	**Uncorrected HR** **(95% CI)**	**Corrected HR** **(95% CI)**	**Uncorrected HR** **(95% CI)**	**Corrected HR** **(95% CI)**	**Uncorrected HR** **(95% CI)**	**Corrected HR** **(95% CI)**

None	1.24 (1.16–1.33)	1.36 (1.23–1.50)	1.18 (1.06–1.31)	1.27 (1.09–1.47)	1.35 (1.20–1.52)	1.54 (1.30–1.82)	1.34 (1.18–1.52)	1.52 (1.26–1.82)
Age, field center	1.25 (1.17–1.34)	1.39 (1.28–1.54)	1.20 (1.08–1.33)	1.30 (1.12–1.51)	1.34 (1.19–1.50)	1.52 (1.29–1.79)	1.32 (1.17–1.50)	1.49 (1.25–1.78)
Age, field center, LDL-C, HDL-C, diabetes, smoking status	1.17 (1.09–1.26)	1.27 (1.14–1.40)	1.14 (1.03–1.25)	1.20 (1.04–1.39)	1.27 (1.13–1.43)	1.41 (1.19–1.67)	1.20 (1.07–1.35)	1.31 (1.11–1.54)
Age, field center, LDL-C, HDL-C, diabetes, smoking status, hypertension	1.15 (1.08–1.24)	1.24 (1.12–1.37)	1.11 (1.01–1.23)	1.16 (1.01–1.35)	1.25 (1.11–1.41)	1.39 (1.17–1.65)	1.20 (1.07–1.34)	1.30 (1.10–1.53)

## References

[B1] Greenland P, Abrams J, Aurigemma GP, Bond MG, Clark LT, Criqui MH, Crouse JR, Friedman L, Fuster V, Herrington DM, Kuller LH, Ridker PM, Roberts WC, Stanford W, Stone N, Swan J, Taubert KA, Wexler L (2000). Prevention Conference V. Beyond secondary prevention: identifying the high-risk patient for primary prevention. Noninvasive tests of atherosclerotic burden. Circulation.

[B2] Ogren M, Hedblad B, Isacsson SO, Janzon L, Jungquist G, Lindell SE (1993). Non-invasively detected carotid stenosis and ischaemic heart disease in men with leg arteriosclerosis. Lancet.

[B3] Leng GC, Fowkes FG, Lee AJ, Dunbar J, Housley E, Ruckley CV (1996). Use of ankle brachial pressure index to predict cardiovascular events and death: a cohort study. BMJ.

[B4] Newman AB, Shemanski L, Manolio TA, Cushman M, Mittelmark M, Polak JF, Powe NR, Siscovick D (1999). Ankle-arm index as a predictor of cardiovascular disease and mortality in the Cardiovascular Health Study. Arteriosclerosis, Thrombosis & Vascular Biology.

[B5] National Cholesterol Education P (2002). Third report of the National Cholesterol Education Program (NCEP) Expert Panel on detection, evaluation, and treatment of high blood cholesterol in adults (Adult Treatment Panel III).  Final report..

[B6] Zheng ZJ, Sharrett AR, Chambless LE, Rosamond WD, Nieto FJ, Sheps DS, Dobs A, Evans GW, Heiss G (1997). Associations of ankle-brachial index with clinical coronary heart disease, stroke and preclinical carotid and popliteal atherosclerosis: the Atherosclerosis Risk in Communities (ARIC) Study. Atherosclerosis.

[B7] Meijer WT, Hoes AW, Rutgers D, Bots ML, Hofman A, Grobbee DE (1998). Peripheral arterial disease in the elderly: The Rotterdam Study. Arteriosclerosis, Thrombosis & Vascular Biology.

[B8] Bots ML, Hofman A, Grobbee DE (1994). Common carotid intima-media thickness and lower extremity arterial atherosclerosis: The Rotterdam Study. Arteriosclerosis & Thrombosis.

[B9] Stoffers HEJH, Rinkens PE, Kester AD, Kaiser V, Knottnerus JA (1996). The prevalence of asymptomatic and unrecognized peripheral arterial occlusive disease. International Journal of Epidemiology.

[B10] Manolio TA, Burke GL, Psaty BM, Newman AB, Haan M, Powe N, Tracy RP, O'Leary DH (1995). Black-white differences in subclinical cardiovascular disease among older adults: the Cardiovascular Health Study. CHS Collaborative Research Group. Journal of Clinical Epidemiology.

[B11] Gofin R, Kark JD, Friedlander Y, Lewis BS, Witt H, Stein Y, Gotsman MS (1987). Peripheral vascular disease in a middle-aged population sample: The Jerusalem Lipid Research Clinic Prevalence Study. Isr J Med Sci.

[B12] Schroll M, Munck O (1981). Estimation of peripheral arteriosclerotic disease by ankle blood pressure measurements in a population study of 60 year old men and women. J Chronic Dis.

[B13] Pomrehn P, Duncan B, Weissfeld L, Wallace RB, Barnes R, Heiss G, Ekelund LG, Criqui MH, Johnson N, Chambless LE (1986). The association of dyslipoproteinemia with symptoms and signs of peripheral arterial disease: The Lipid Research Clinic Prevalence Study. Circulation.

[B14] Agner E (1981). Natural history of angina pectoris, possible previous myocardial infarction and intermittent claudication during the eighth decade. Acta Med Scand.

[B15] Kannel WB, Skinner JJ, Schwartz MJ, Shurtleff D (1970). Intermittent claudication: incidence in the Framingham Study. Circulation.

[B16] Kannel WB, McGhee DL (1985). Update on some epidemiological features of intermittent claudication: the Framingham Study. J Am Ger Soc.

[B17] Leng GC, Lee AJ, Fowkes FG, Whiteman M, Dunbar J, Housley E, Ruckley CV (1996). Incidence, natural history and cardiovascular events in symptomatic and asymptomatic peripheral arterial disease in the general population. International Journal of Epidemiology.

[B18] Hiatt WR, Hoag S, Harmnan RF (1995). Effect of diagnostic criteria on the prevalence of peripheral arterial disease. The San Luis Valley Diabetes Study. Circulation.

[B19] Fowkes FG, Housley E, Riemersma RA, Macintyre CC, Cawood EH, Prescott RJ, Ruckley CV (1992). Smoking, lipids, glucose intolerance, and blood pressure as risk factors for peripheral atherosclerosis compared with ischemic heart disease in the Edinburgh Artery Study. American Journal of Epidemiology.

[B20] Vogt MT, Cauley JA, Newman AB, Kuller LH, Hulley SB (1993). Decreased ankle/arm blood pressure index and mortality in elderly women. JAMA.

[B21] Abbott RD, Petrovitch H, Rodriguez BL, Yano K, Schatz IJ, Popper JS, Masaki KH, Ross GW, Curb JD (2000). Ankle/brachial blood pressure in men >70 years of age and the risk of coronary heart disease. Am J Cardiol.

[B22] Hooi JD, Stoffers HE, Kester AD, van RJ, Knottnerus JA (2002). Peripheral arterial occlusive disease: prognostic value of signs, symptoms, and the ankle-brachial pressure index. Med Decis Making.

[B23] Resnick HE, Lindsay RS, McDermott MM, Devereux RB, Jones KL, Fabsitz RR, Howard BV (2004). Relationship of high and low ankle brachial index to all-cause and cardiovascular disease mortality: the Strong Heart Study. Circulation.

[B24] O'Hare AM, Katz R, Shlipak MG, Cushman M, Newman AB (2006). Mortality and cardiovascular risk across the ankle-arm index spectrum. Circulation.

[B25] Wattanakit K, Folsom AR, Duprez DA, Weatherley BD, Hirsch AT (2006). Clinical significance of a high ankle-brachial index: Insights from the Atherosclerosis Risk in Communities (ARIC) Study. Atherosclerosis.

[B26] Jones DW, Chambless LE, Folsom ARJ, Heiss G, Hutchinson RG, Sharrett AR, Szklo M, Taylor HA (2002). Risk factors for coronary heart disease in African Americans: the Atherosclerosis Risk in Communities Study, 1987-1997. Arch Int Med.

[B27] Jackson SA, Burke GL, Thach C, Cushman M, Ives D, Powe N, Manolio TA (2001). Incidence and predictors of coronary heart disease among older African Americans--the Cardiovascular Health Study. J Natl Med Assoc.

[B28] (1989). The Atherosclerosis Risk in Communities (ARIC) Study: design and objectives. American Journal of Epidemiology.

[B29] Jackson R, Chambless L, Yang K, Byrne T, Watson R, Folsom A, Shahar E, Kalsbeek W (1996). Differences between respondents and non-respondents in a multi-center community-based study vary by gender and ethnicity. J Clin Epidemiol.

[B30] Orchard TJ, Strandness DE (1993). Assessment of peripheral vascular disease in diabetes. Report and recommendations of an international workshop sponsored by the American Diabetes Association and the American Heart Association. September 18-20, 1992, New Orleans, Louisiana. Circulation.

[B31] Weatherley BD, Chambless LE, Heiss G, Catellier DJ, Ellison RC (2006). The reliability of the ankle-brachial index in the Atherosclerosis Risk in Communities (ARIC) study and the NHLBI Family Heart Study (FHS). BMC Cardiovascular Disorders.

[B32] Friedewald WT, Levy RI, Frederickson DS (1972). Estimation of the concentration of low-density lipoprotein cholesterol in plasma, without use of preparative centrifuge. Clin Chem.

[B33] Rose GA, Blackburn H (1968). Cardiovascular Survey Methods.

[B34] Riley WA, Barnes RW, Bond MG, Evans G, Chambless LE, Heiss G (1991). High-resolution B-mode ultrasound reading methods in the Atherosclerosis Risk in Communities (ARIC) Cohort. J Neuroimag.

[B35] Rautaharju PM, Warren JW, Jain U, Wolf HK, Nielsen CL (1981). Cardiac infarction injury score: an electrocardiographic coding scheme for ischemic heart disease. Circulation.

[B36] Schoenfeld DA (1982 ). Partial residuals for the proportional hazards regression model. Biometrika.

[B37] Stone CJ, Koo CY (1985 ). Additive splines in statistics. Proc Stat Comp Sect Am Statist Assoc.

[B38] Harrell FE (1995 ). Predicting Outcomes: Applied Survival Analysis and Logistic Regression.

[B39] Fuller WA (1987). Measurement error models.

[B40] Carroll RJ, Ruppert D, Stefanski LA (1995). Measurement error in nonlinear models.

[B41] Whittemore AS (1989). Errors-in-variables regression using Stein estimates. The American Statistician.

[B42] Heald CL, Fowkes FG, Murray GD, Price JF (2006). Risk of mortality and cardiovascular disease associated with the ankle-brachial index: Systematic review. Atherosclerosis.

[B43] Ogren M, Hedblad B, Jungquist G, Isacsson SO, Lindell SE, Janzon L (1993). Low ankle-brachial pressure index in 68-year-old men: prevalence, risk factors and prognosis. Results from prospective population study "Men born in 1914", Malmo, Sweden. Eur J Vasc Surg.

[B44] Murabito JM, Evans JC, Larson MG, Nieto K, Levy D, Wilson PWF (2003). The ankle-brachial index in the lederly and risk of stroke, coronary disease, and death: the Framingham Study. Arch Int Med.

[B45] Lee AJ, Price JF, Russell MJ, Smith FB, van Wijk MC, Fowkes FG (2004). Improved prediction of fatal myocardial infarction using the ankle brachial index in addition to conventional risk factors: the Edinburgh Artery Study. Circulation.

[B46] van der Meer IM, Bots ML, Hofman A, del Sol AI, van der Kuip DA, Witteman JC (2004). Predictive value of noninvasive measures of atherosclerosis for incident myocardial infarction: the Rotterdam Study. Circulation.

[B47] Aronow WS (1992). Prevalence of atherothrombotic brain infarction, coronary artery disease and peripheral arterial disease in elderly blacks, Hispanics and whites. Am J Cardiol.

[B48] Newman AB, Siscovick DS, Manolio TA, Polak J, Fried LP, Borhani NO, Wolfson SK (1993). Ankle-arm index as a marker of atherosclerosis in the Cardiovascular Health Study. Circulation.

[B49] Kullo IJ, Bailey KR, Kardia SL, Mosley TH, Boerwinkle E, Turner ST (2003). Ethnic differences in peripheral arterial disease in the NHLBI Genetic Epidemiology Network of Arteriopathy (GENOA) study. Vasc Med.

[B50] Selvin E, Erlinger TP (2004). Prevalence of and risk factors for peripheral arterial disease in the United States: results from the National Health and Nutrition Examination Survey, 1999-2000. Circulation.

[B51] McDermott MM, Liu K, Criqui MH, Ruth K, Goff D, Saad MF, Wu C, Homma S, Sharrett AR (2005). Ankle-brachial index and subclinical cardiac and carotid disease: the multi-ethnic study of atherosclerosis. Am J Epidemiol.

[B52] Collins TC, Petersen NJ, Suarez-Almazor M, Ashton CM (2003). The prevalence of peripheral arterial disease in a racially diverse population. Arch Intern Med.

[B53] Wang JC, Criqui MH, Denenberg JO, McDermott MM, Golomb BA, Fronek A (2005 ). Exertional leg pain in patients with and without peripheral arterial disease. Circulation.

[B54] Fowkes FG, Housley E, Macintyre CC, Prescott RJ, Ruckley CV (1988). Variability of ankle and brachial systolic pressures in the measurement of atherosclerotic peripheral arterial disease. Journal of Epidemiology & Community Health.

[B55] Flegal CM, Keyl PM, Nieto FJ (1991). Differential misclassification arising from nondifferential errors in exposure measurement. American Journal of Epidemiology.

